# Longitudinal Study of Spatially Heterogeneous Emphysema Progression in Current Smokers with Chronic Obstructive Pulmonary Disease

**DOI:** 10.1371/journal.pone.0044993

**Published:** 2012-09-18

**Authors:** Naoya Tanabe, Shigeo Muro, Susumu Sato, Shiro Tanaka, Tsuyoshi Oguma, Hirofumi Kiyokawa, Tamaki Takahashi, Daisuke Kinose, Yuma Hoshino, Takeshi Kubo, Toyohiro Hirai, Michiaki Mishima

**Affiliations:** 1 Department of Respiratory Medicine, Graduate School of Medicine, Kyoto University, Kyoto, Japan; 2 Division of Clinical Trial Design and Management, Translational Research Center, Kyoto University, Kyoto, Japan; 3 Department of Diagnostic Imaging and Nuclear Medicine, Kyoto University, Kyoto, Japan; Clinica Universidad de Navarra, Spain

## Abstract

**Background:**

Cigarette smoke is the main risk factor for emphysema, which is a key pathology in chronic obstructive pulmonary disease (COPD). Low attenuation areas (LAA) in computed tomography (CT) images reflect emphysema, and the cumulative size distribution of LAA clusters follows a power law characterized by the exponent D. This property of LAA clusters can be explained by model simulation, where mechanical force breaks alveolar walls causing local heterogeneous lung tissue destruction. However, a longitudinal CT study has not investigated whether continuous smoking causes the spatially heterogeneous progression of emphysema.

**Methods:**

We measured annual changes in ratios of LAA (LAA%), D and numbers of LAA clusters (LAN) in CT images acquired at intervals of ≥3 years from 22 current and 31 former smokers with COPD to assess emphysema progression. We constructed model simulations using CT images to morphologically interpret changes in current smokers.

**Results:**

D was decreased in current and former smokers, whereas LAA% and LAN were increased only in current smokers. The annual changes in LAA%, D, and LAN were greater in current, than in former smokers (1.03 vs. 0.37%, p = 0.008; −0.045 vs. −0.01, p = 0.004; 13.9 vs. 1.1, p = 0.007, respectively). When LAA% increased in model simulations, the coalescence of neighboring LAA clusters decreased D, but the combination of changes in D and LAN in current smokers could not be explained by the homogeneous emphysema progression model despite cluster coalescence. Conversely, a model in which LAAs heterogeneously increased and LAA clusters merged somewhat in relatively advanced emphysematous regions could reflect actual changes.

**Conclusions:**

Susceptibility to parenchymal destruction induced by continuous smoking is not uniform over the lung, but might be higher in local regions of relatively advanced emphysema. These could result in the spatially heterogeneous progression of emphysema in current smokers.

## Introduction

Chronic obstructive pulmonary disease (COPD) is a leading cause of death and it imposes increasing economic and social burdens [1]. Emphysema is a major pathological change of COPD that is characterized by the abnormal and permanent enlargement of distal airspaces and alveolar wall destruction [2]. It causes airflow limitations [3], impaired diffusion capacity [4] and increased mortality [5], [6]. Therefore, understanding the mechanism of emphysema progression is important to improve COPD management.

Cigarette smoke is a major risk factor in the development and progression of emphysema [1]. It induces inflammation, which causes excessive oxidative stress, a protease anti-protease imbalance, epithelial cell apoptosis and extracellular matrix remodeling, resulting in emphysema induction [1], [7], [8], [9], [10]. Although these components might affect the characteristic structural alterations of emphysema, the spatial and morphological properties of cigarette smoke-induced emphysema progression are not completely understood, particularly in humans.

In addition to direct damage caused by inhaled cigarette smoke, the role of mechanical force should be considered in lung parenchymal destruction. Mechanical force is generated against the alveolar walls during breathing cycles with intermittent deep inspiration, and can be concentrated along with weakening of the walls during emphysematous changes [11]. Alveolar walls weakened by proteolysis in animal models of elastase-induced emphysema can be ruptured by mechanical force [12], [13]. Furthermore, mechanical forces accelerate elastin cleavage by elastase and increase the available binding sites on elastin in the lung [14]. Computer simulation analysis has shown that force is redistributed among neighboring walls, and some neighboring areas receive increased force when an alveolar wall fails [15]. Therefore, mechanical force can cause the local propagation of tissue destruction and increase the spatial heterogeneity of emphysema [16].

Computed tomography (CT) of the chest is an important tool with which to assess longitudinal structural alterations associated with emphysema in humans [17], [18]. Low attenuation areas (LAA) in CT images reflect pathological emphysema and the ratio (%) of LAA to the total lung volume (LAA%) is a standard index of the extent of emphysema [19], [20]. The cumulative size distribution of LAA clusters follows a power law characterized by the exponent D [4]. The values of D reflect the fractal dimension of terminal airspace geometry and sensitively detect alveolar tissue destruction. D values can provide additional information about the morphological features of emphysema [4], [17], [21]. We previously found that D is smaller in patients with mild COPD than in healthy individuals even when LAA% does not differ, and that the numbers of smaller and larger LAA clusters are decreased and increased, respectively, in patients with COPD whereas healthy individuals have many small clusters [4].

A previous simulation analysis found that these changes in the size and number of LAA clusters cannot be explained using a model of diffuse alveolar wall destruction, but a model of mechanical force-based destruction is in agreement with the actual properties of clusters in patients with COPD [16]. Such destruction is closely associated with the coalescence of LAA clusters, which can be detected as a decrease in the value of D [4]. Notably, since mechanical force can be concentrated in already damaged regions [15], [16], the simulation analysis led to the hypothesis that alveolar wall rupture is probably not uniform in the lung, and might be more severe in local regions of relatively advanced emphysema. With respect to the influence of continuous smoking, CT studies have shown that annual changes in LAA% and the number of LAA clusters (LAN) are greater in current, than in former smokers [22], [23]. However, a longitudinal study has not yet investigated whether continuous smoking induces further emphysema progression predominantly in areas that are already quite damaged.

We hypothesized that local regions with relatively advanced emphysematous change are prone to further parenchymal destruction, and that continuous smoking thus causes the spatially heterogeneous progression of emphysema. The present study evaluates annual changes in LAA%, D, and LAN using CT in current and former smokers with mild to moderate emphysema. Annual changes in LAA%, D, and LAN were greater in current, than former smokers. We also performed model simulations using CT images to interpret the results and found that the actual changes in LAA%, D, and LAN in current smokers cannot be explained by a model in which uniform susceptibility to parenchymal destruction over the lung leads to spatially homogeneous emphysema progression. However, these changes were reflected by another model that leads to spatially heterogeneous increases in LAAs and the coalescence of LAA clusters to some extent in relatively advanced emphysematous regions.

## Methods

### Ethics Statement

The ethics committee of Kyoto University approved the study (approval No. E182) and all patients provided written informed consent to participate.

### Study Protocol

**Figure 1:**
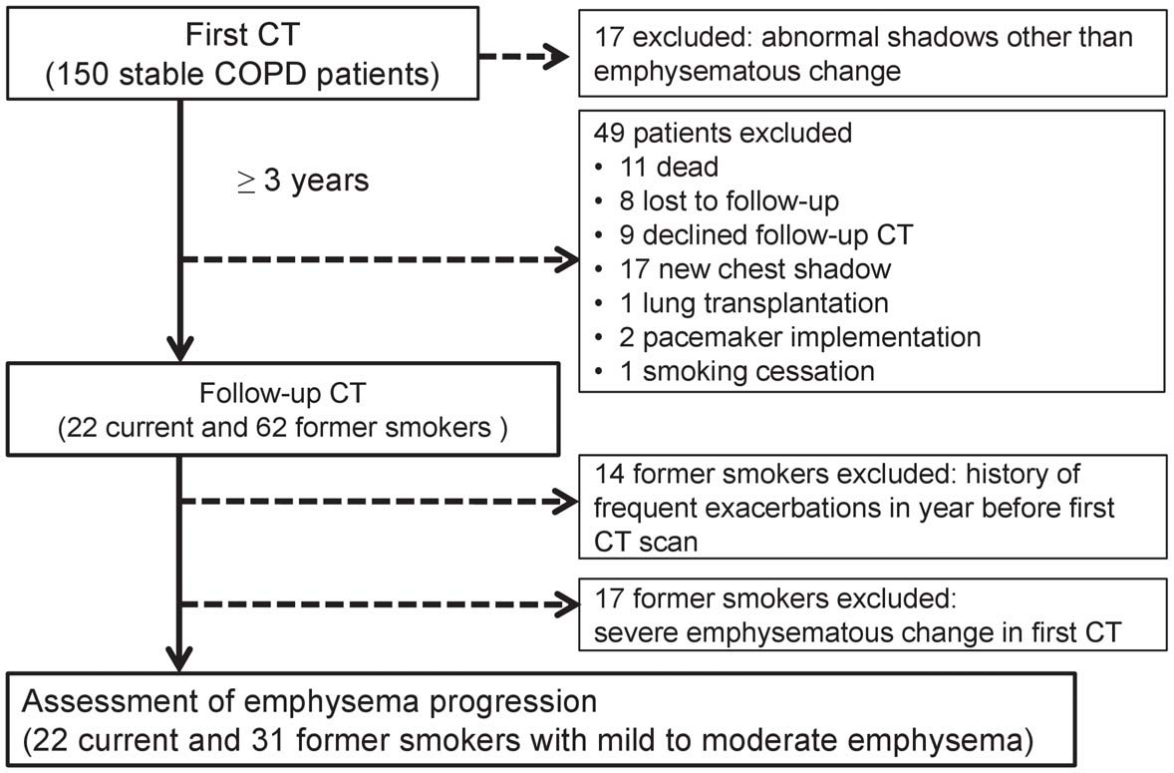
Study design and flow of patients through the study.

**Figure 2:**
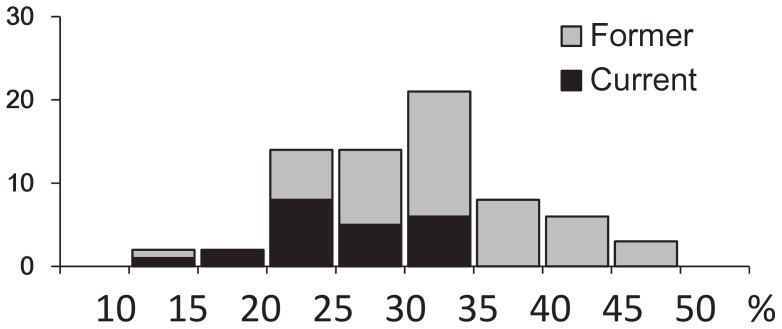
Histogram of emphysema severity. LAA% was assessed in 70 patients with COPD. LAA% in all current smokers was <35%. Severe emphysema was defined as ≥35% of LAA%, and former smokers with severe emphysema were excluded from the study.

This is part of an ongoing COPD observational study at Kyoto University. The study protocol is summarized in Figure 1. We examined 150 patients with COPD who had been followed up for at least one year at Kyoto University Hospital using high-resolution chest CT between April 2006 and June 2008. Among them, 17 with abnormal CT findings other than emphysematous change were excluded. The remaining patients were followed up by evaluating longitudinal changes in CT parameters and pulmonary function over at least three years. During the follow-up, 49 were excluded because of death, loss to follow-up, withdrawal of consent, appearance of new shadows on chest images, lung transplantation, pacemaker implementation, or smoking cessation. Thus, 84 patients underwent follow-up CT evaluation. Among them, 14 former smokers had experienced at least two exacerbations in the year before the first CT scan, but the current smokers had none. We excluded these former smokers in assessing emphysema progression, because a history of frequent exacerbation is a powerful predictor of future exacerbation [24] that is associated with emphysema progression [17]. Exacerbation was defined as symptomatic deterioration requiring antibiotics and/or systemic corticosteroid. Whole-lung emphysema severity was evaluated from the initial CT images of the remaining 70 patients (Figure 2). Since the emphysema severity assessed as LAA% in all current smokers was mild to moderate (LAA% <35%), 17 former smokers with severe emphysematous change (LAA% ≥ 35%) were excluded. Therefore, our final study population comprised 53 patients, of whom 22 and 31 were current and former smokers, respectively.

### Smoking Status, Pulmonary Function Tests, and CT Acquisition

Smoking status was determined based on self-reporting. Levels of carboxyhemoglobin (COHb) in arterial blood were measured to confirm the validity of smoking cessation reported by patients. Patients were examined using a high-resolution CT (Aquilion 64; Toshiba; Tokyo, Japan) with a slice thickness of 0.5 mm and by pulmonary function tests (PFTs) (Chestac-65V; Chest MI Corp; Tokyo, Japan) during exacerbation-free periods as described [17], [25]. Multi-detector CT images were acquired using 0.5-mm collimation, a scan time of 500 milliseconds, 120 kV peak and auto exposure control, and then the data were reconstructed using a sharp kernel (FC56). We used only one CT scanner to avoid inter-scanner variability and a custom-designed application to analyze the CT parameters [17]. In addition to routine calibration using an air and water phantom, CT numbers were corrected using tracheal air density [17]. The trachea was identified with a threshold of −800 HU, the area of the lumen (Atr) was measured, and the mean radius (Rtr) of the lumen was calculated. The mean CT number of the region within a circle with a half mean radius (Rtr/2) from the gravity center of the lumen (CTair) was calculated. All the original CT numbers were then corrected for each patients using the formula: corrected CT number = (−1000× original CT number)/CTair.

### Analysis of Emphysematous Changes and Lung Volumes

We calculated LAA% using a threshold of −960 HU as described [17], [25] to assess emphysematous change [19], [20] and total lung volume (CT-TLV). We also analyzed LAA clusters as follows. The cumulative frequency distribution of the size of LAA clusters Y, which was defined as the number of clusters that are larger than the size of a given cluster X, can be described by a power law of X of the form: Y = K × X^−D^ [4]. The values of D were obtained by linear regression and calculated as the slope of the straight line in log-log plots. We also measured the number of LAA clusters (LAN) in each slice from the entire lung and averaged them to determine the value of LAN in the whole lung as described [23].

### Model Simulation

**Figure 3:**
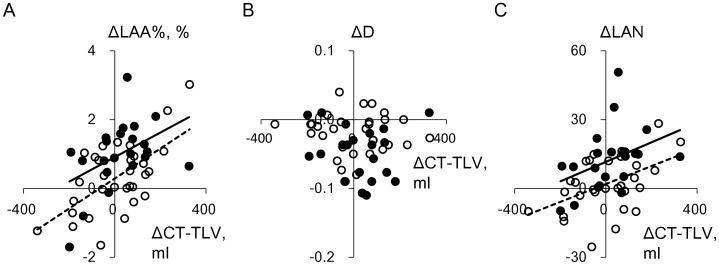
Schematic explanation of model simulation. Representative CT images from current smokers are shown as binary images, in which black and gray represent pixels with low attenuation and normal density, respectively (A). One pixel was selected from all pixels with normal density and changed to new low attenuation according to two models. Probabilities of selecting pixels between extant low attenuation clusters leading to their coalescence were determined in advance for both models. According to coalescence rates, pixels with normal density were changed into new pixels with low attenuation leading to coalescence or appearance/enlargement of low attenuation clusters (yellow and green pixels, respectively). New, low-attenuation pixels were randomly selected from among all pixels with normal density in the random model (B). Normal density pixels were identified (C) among local advanced emphysematous lesions (blue pixels) in the damage-dependent model, and then new low attenuation pixels were randomly selected from these relatively damaged local lesions (D).

To determine whether emphysema progresses in relatively damaged regions or diffusely over the lungs of current smokers, we performed model simulation using seven representative CT images of the initial CT evaluations from seven current smokers as described with slight modification [17]. Figure 3 shows that we selected one pixel with a normal density (ND) and changed it into a new LAA pixel. Since changing ND pixels separating adjoining LAA clusters into new LAA pixels causes LAA clusters to coalescence, which reduces D, the probability of selecting these pixels leading to LAA cluster coalescence was set at 0, 15, or 30% in advance.

We also established two new models in which a new LAA pixel was randomly selected from all ND pixels (random model, Figure 3B) or from ND pixels in regions of relatively advanced emphysema (damage-dependent model, Figure 3C and D). The random model simulated spatially homogeneous emphysema progression where the susceptibility to parenchymal destruction induced by continuous smoking was uniform over the lung, whereas the damage-dependent model simulated spatially heterogeneous emphysema progression where local regions of relatively advanced emphysema were prone to parenchymal destruction. We initially determined which ND pixels were located in regions of relatively advanced emphysema in the damage-dependent model. We set a circular region of interest (ROI) on each ND pixel with a radius of three pixels and then measured the local LAA% in the ROI. When the local LAA% was higher than that in the total lung in CT images, we considered that this ND pixel was in a region of relatively advanced emphysema.

Collectively, the present simulations were performed using six algorithms, which consisted of random and damage-dependent models each with 0, 15, or 30% coalescence. We iteratively repeated changing a ND pixel into a new LAA pixel for each algorithm using the modified image as the starting point for the next selection until the increase in LAA% reached 1, 2 or 3% from baseline, and then D and LAN were measured using the modified image. We performed twenty simulations to measure the changes in D and LAN and averaged the twenty sets of the changes in D and LAN. Finally, the mean changes in D and LAN in seven CT images were calculated as a representative value of each algorithm.

### Statistical Analyses

Data were statistically analyzed using JMP 7 software (SAS Institute, Cary, NC, USA) and are expressed as medians (25th and 75th percentiles). Annual changes in CT and pulmonary function parameters between and within groups were analyzed using the Mann-Whitney U and Wilcoxon signed-rank tests, respectively. Normality of data was assessed graphically and by the Shapiro-Wilks normality test. It was found that baseline D, LAN, and CT-TLV and annual changes in LAA%, D, LAN, and CT-TLV were normally distributed while baseline LAA% was not. Thus, in addition to the natural values of LAA%, log-transformed LAA% (LogLAA%) was used in statistical analysis including baseline CT parameters of emphysema. Multivariate regression analysis were also performed with annual changes in CT parameters of emphysema as dependent variables, and baseline CT parameters, smoking status, and changes in CT-TLV as independent variables. After confirming normal distributions of the annual changes in LAA%, D, LAN, and CT-TLV, we tested correlations among these changes separately in current and former smokers using the Pearson correlation test, and then whether smoking status can affect the association between changes in LAA% and D or between those in LAA% and LAN was investigated by assessing the effects of interactions between changes in LAA% and smoking status (current vs. former) on changes in D or LAN, respectively. All *p-*values are presented as two-sided and *p*<0.05 was considered significant.

## Results

### Characteristics of Patients

The baseline characteristics of the 22 current and 31 former smokers are shown in Table 1. Age, sex, body mass index, smoking history, forced expiratory volume in one second (FEV_1_), FEV_1%_ predicted (%FEV_1_), Global Initiative for Chronic Obstructive Lung Disease (GOLD) stage, LAA%, LogLAA%, D, LAN and CT-TLV did not differ between the two groups. Use of regular COPD medications including tiotropium, a long-acting beta2 agonist, and inhaled corticosteroid did not differ. Levels of COHb were higher in the current, than in the former smokers. The median follow-up periods for the former and current smokers were 3.58 and 3.51 years, respectively, and did not significantly differ between the two groups.

**Table 1:** Characteristics of study patients at baseline computed tomography scan (n = 53).

	Former (n = 31)		Current (n = 22)		*p* value
Age (years)	70	(60.0, 75.0)	65.5	(62.0, 72.3)	0.54
Sex (male:female)	28∶3		20∶2		0.94
Body mass index	22.7	(21.3, 24.2)	21.3	(19.6, 22.9)	0.06
Smoking history (pack-years)	50.0	(40.0, 94.0)	59.0	(45.5, 99.0)	0.36
Blood COHb (%)*	1.0	(0.7, 1.2)	2.6	(1.8, 3.8)	<0.0001
FEV_1_ (L)	1.94	(1.50, 2.59)	1.96	(1.25, 2.47)	0.75
%FEV_1_ (%)	71.0	(57.5, 88.0)	71.2	(53.8, 81.5)	0.34
GOLD stage (I/II/III/IV)	10/19/2/0		8/10/5/0		0.21
LAA%	29.7	(25.0, 31.5)	25.8	(22.7, 30.1)	0.08
LogLAA%	1.47	(1.40, 1.50)	1.41	(1.36, 1.48)	0.08
D	1.80	(1.55, 2.02)	1.83	(1.67, 2.32)	0.25
LAN	137	(84, 195)	123	(61, 197)	0.30
CT-TLV (L)	5.02	(4.25, 5.42)	4.84	(4.26, 6.08)	0.57
Tiotropium (%)†	6.5		9.0		0.72
Long-acting beta2-agonist (%)†	9.7		4.5		0.47
Inhaled corticosteroid (%)†	19.4		9.0		0.09
Salmeterol/fluticasone combination (%)†	0		0		1.00

Data are expressed as medians (25th and 75th percentiles). Abbreviations: COHb, carboxyhemoglobin; CT-TLV, total lung volume measured by computed tomography; Current, current smokers; D, index of low attenuation cluster analysis; FEV_1_, forced expiratory volume in one second; %FEV_1_, FEV_1%_ predicted; Former, former smokers; GOLD, Global Initiative for Chronic Obstructive Lung Disease;

LAA%, ratio (%) of low attenuation area; LAN; number of low attenuation clusters.

* Blood COHb was not measured in one current smoker.

† Ratio (%) of patients prescribed with these drugs.

### Annual Changes in Pulmonary Function and CT Parameters

As shown in Table 2, the FEV_1_ and D significantly decreased in both groups during the follow-up, whereas changes in CT-TLV were not significant in either. The LAA%, LogLAA%, and LAN significantly increased in current smokers, but did not significantly change in former smokers. The annual changes in FEV_1_ and CT-TLV did not differ between the two groups. The annual changes in LAA%, LogLAA%, D, and LAN were significantly greater in current, than in former smokers (1.03 vs. 0.37% per year, p = 0.008; 0.02 vs. 0.01 per year, p = 0.007; −0.045 vs. −0.01 per year, p = 0.004; 1.1 vs. 13.9, p = 0.007, respectively).

**Table 2:** Annual changes in lung function and computed tomography parameters in current and former smokers (n = 53).

	Former (n = 31)		Current (n = 22)		*p* ^†^
FEV_1_, mL	−40.0	(−83.7, −12.6)	−74.5	(−83.8, −21.4)	0.27
p*		0.001		<0.0001	
LAA%	0.37	(−0.37, 0.93)	1.03	(0.67, 1.5)	0.008
*p* *		0.07		0.0002	
LogLAA%	0.01	(0.01, 0.02)	0.02	(−0.01, 0.02)	0.007
*p* *		0.07		0.0007	
D	−0.01	(−0.04, 0)	−0.045	(−0.09, −0.018)	0.004
p*		0.0006		<0.0001	
LAN	1.1	(−3.1, 12.0)	13.9	(4.1, 15.8)	0.007
p*		0.29		<0.0001	
CT-TLV, mL	42.9	(−87.0, 83.8)	30.5	(−41.2, 96.2)	0.75
p*		0.70		0.48	

p* intra-group;

*p* ^†^ inter-group. Data are expressed as medians (25th and 75th percentiles). Abbreviations: CT-TLV, total lung volume measured by computed tomography; Current, current smokers; D, index of low attenuation cluster analysis; FEV_1_, forced expiratory volume in one second; Former, former smokers; LAA%, ratio (%) of low attenuation area; LAN; number of low attenuation clusters.

The results of the multivariate regression analysis in Table 3 show that continuous smoking was independently associated with the increases in LAA% and LAN, and the decrease in D (p = 0.02, 0.006, 0.002, respectively) after adjustment for baseline CT parameters of emphysema and changes in CT-TLV. An independent association between continuous smoking and annual change in LogLAA% was also found in the model with log transformed LAA% (p = 0.01).

**Table 3:** Multivariate regression analysis of relative contribution of each variable to predict annual changes in CT parameters of emphysema (n = 53).

	Estimate	95% Confidence interval	*p* value
**Change in LAA%**			
Continuous smoking, yes/no	0.61	(0.12 to 1.09)	0.02
Change in CT-TLV/1 mL increase	0.004	(0.002 to 0.006)	<0.0001
Baseline LAA%/1% increase	−0.0004	(−0.047 to 0.046)	0.99
**Change in LogLAA%**			
Continuous smoking, yes/no	0.0094	(0.0021 to 0.168)	0.01
Change in CT-TLV/1 mL increase	0.0001	(0.0000 to 0.0001)	<0.0001
Baseline LogLAA%/increase of 1	−0.01	(−0.049 to 0.030)	0.62
**Change in D**			
Continuous smoking, yes/no	−0.030	(−0.049 to −0.011)	0.002
Change in CT-TLV/1 mL increase	0.000	(0.000 to 0.000)	0.48
Baseline D/increase of 1	−0.0043	(−0.030 to 0.022)	0.73
**Change in LAN**			
Continuous smoking, yes/no	9.38	(0.011 to 0.082)	0.006
Change in CT-TLV/1 mL increase	0.041	(0.017 to 0.064)	0.001
Baseline LAN/increase of 1	−0.014	(−0.056 to 0.037)	0.54

Abbreviations: CT-TLV, total lung volume measured by computed tomography; D, index of low attenuation cluster analysis; LAA%, ratio (%) of low attenuation area; LAN, number of low attenuation clusters.

**Figure 4:**
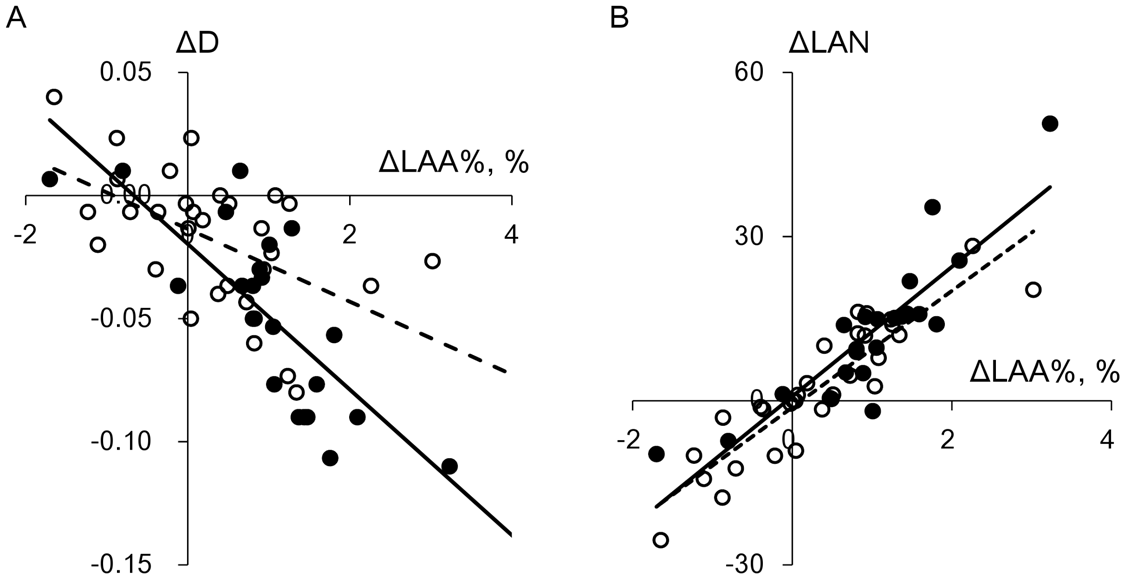
Relationships between annual changes in lung volume and emphysema parameters. A, B, and C show relationships between annual changes in CT-TLV and LAA%, D, and LAN, respectively, in 22 current and 31 former smokers with mild to moderate emphysema. Closed and open circles, current and former smokers, respectively. Solid and dashed regression lines, current and former smokers, respectively. Changes in CT-TLV in current and former smokers significantly correlated with those in LAA% (r = 0.45, p = 0.03 and r = 0.62, p = 0.002, respectively), but not in D (r = −0.11, p = 0.64, and r = −0.08, p = 0.66, respectively). Changes in LAN and CT-TLV significantly correlated in former smokers, and tended to correlate in current smokers (r = 0.47, p = 0.008, and r = 0.40, p = 0.06, respectively).

**Figure 5:**
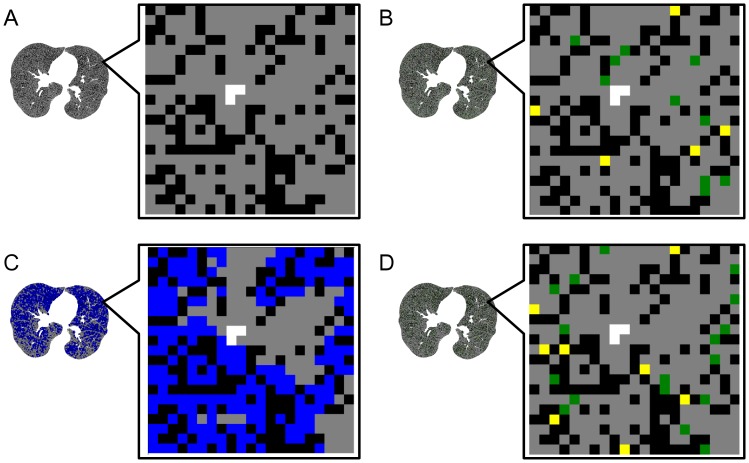
Relationships among annual changes in CT parameters of emphysema. A and B show relationships between the changes in LAA% and D and between those in LAA% and LAN, respectively, in 22 current and 31 former smokers with mild to moderate emphysema. Closed and open circles, current and former smokers, respectively. Solid and dashed regression lines, current and former smokers, respectively. Changes in LAA% in current and former smokers significantly correlated with those in D (r = −0.77, p<0.0001; r = −0.53, p = 0.002, respectively) and LAN (r = 0.88, p<0.0001 and r = 0.90, p<0.0001, respectively). Smoking status and change in LAA% significantly interacted to elicit changes in D (p = 0.03), but not in LAN (p = 0.48).

Figure 4 shows plots of the changes in LAA%, D, and LAN against those in CT-TLV in current and former smokers. All these changes were normally distributed. The changes in CT-TLV in both groups significantly correlated with those in LAA% (r = 0.45, p = 0.03 and r = 0.62, p = 0.002, respectively), but not with those in D (r = −0.11, p = 0.64, and r = −0.08, p = 0.66, respectively). The changes in LAN and CT-TLV significantly correlated in former smokers, and tended to correlate in current smokers (r = 0.47, p = 0.008, and r = 0.40, p = 0.06, respectively). Figure 5 shows that the changes in LAA% in current and former smokers significantly correlated with those in D (r = −0.77, p<0.0001 and r = −0.53, p = 0.002, respectively) and LAN (r = 0.88, p<0.0001 and r = 0.90, p<0.0001, respectively). Furthermore, the changes in LAA% and smoking status significantly interacted with respect to their effects on changes in D (p = 0.03), but not on those in LAN (p = 0.48).

### Model Simulation

**Figure 6:**
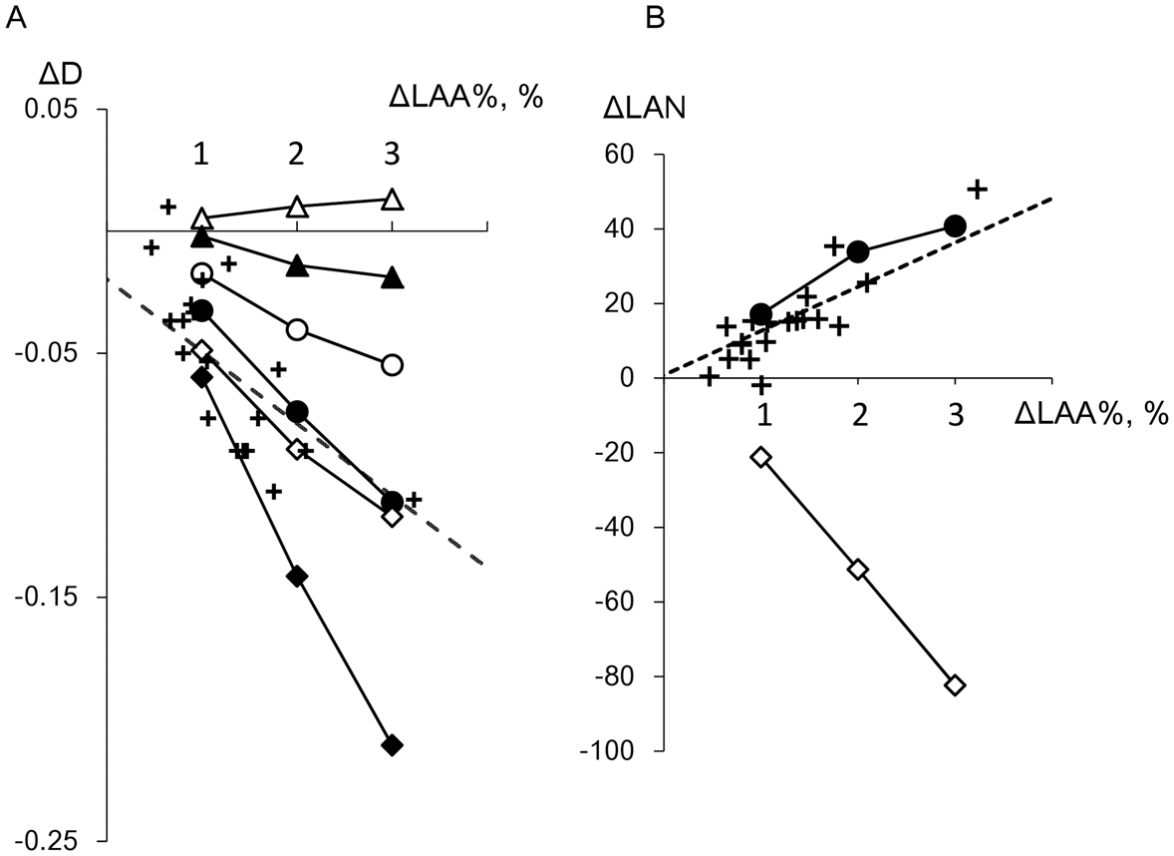
Changes in LAA%, D and LAN determined from model simulations and current smokers. Crosses, current smokers; dashed line, regression calculated from current smokers. Closed and open symbols, random and damage-dependent models, respectively. Triangles, without coalescence of low attenuation clusters; circles and diamonds, 15% and 30% cluster coalescence, respectively. Decreases in D obtained from random and damage-dependent models with 30% and 15% coalescence, respectively, were similar to actual data from current smokers (A). LAN increased when LAA% increased in damage-dependent model with 15% coalescence, which closely agreed with data from current smokers (B).

We supposed that not only the coalescence of LAA clusters, but also the spatially heterogeneous disposition of new LAAs could cause the accelerated decrease in D in current smokers as reported [17]. Model simulations proceeded according to six algorithms, the random and damage-dependent models with 0, 15, and 30% coalescence of adjoining LAA clusters as described in the Methods section. Since D complements LAA% in assessments of emphysema [4], [17], [21], we first determined which algorithms were in good agreement with the actual change in D in current smokers when LAA% increased. The data in Figure 6A show that changes in D were greater in both models along with an increase in the rate of coalescence (0, 15% and 30%). Decreases in D were always greater for any rate of coalescence in the damage-dependent, than in the random model. Consequently, the random and damage-dependent models with 30% and 15% coalescence, respectively, led to decreases in D, which were similar to those obtained from the regression line for current smokers generated from actual data.

Moreover, changes in LAN were also compared among the random and damage-dependent models with 30% and 15% coalescence, respectively, and actual data derived from current smokers. Figure 6B shows that the damage-dependent model with 15% coalescence resulted in an increase in LAN when LAA% increased, which closely agreed with the actual data from current smokers.

## Discussion

Annual changes in CT parameters of emphysema were greater in current, than in former smokers in the present study. The deleterious effect of continuous smoking persisted even after adjustment for baseline CT parameters and changes in lung volume measured by CT. When LAA% increased in the model simulation, the model of spatially homogeneous emphysema progression could not explain the increase in LAN and the decrease in D in current smokers. However, the exclusive dispersion of new LAA pixels in relatively damaged regions leading to spatially heterogeneous progression in another model closely agreed with the actual changes in current smokers. Although experimental models of emphysema and a cross-sectional CT study have indicated that the spatial heterogeneity of emphysema is induced by mechanical force, the findings of the present longitudinally study confirmed that continuous smoking causes the spatially heterogeneous progression of emphysema.

Cigarette smoke causes the proteolysis of extracellular matrix such as collagen and elastin, oxidation, apoptosis of lung epithelial cells, and impairs tissue repair [1], [7], [8], [9], [10]. Inhaled cigarette smoke not only directly ruptures alveolar walls, but also promotes rupture induced by mechanical force through the reduction of alveolar wall strength. A previous computer simulation study found that the actual size distribution of LAA clusters in patients with COPD cannot be explained by a model of random tissue destruction, which might partially reflect direct damage induced by cigarette smoke [4], [16]. That study also showed that destruction due to mechanical force might be required to produce the heterogeneity of cluster sizes and distribution. Since these findings were based on data from both current and former smokers in a cross-sectional study, our longitudinal study investigated whether continuous smoking causes spatially heterogeneous emphysema.

The LAA% and LAN increased while D decreased in current smokers. These findings are consistent with those of a study showing that LAN tended to be increased in current smokers, and that annual changes in LAN as well as LAA% were greater in current, than in former smokers [23].

Smoking status and the change in LAA% significantly interacted with respect to their effect on the change in D, but not LAN. This statistically demonstrated that the slope of the regression line of D on LAA% in figure 5 was steeper in current than in former smokers. This supports the notion that D provides additional information about emphysematous changes. Indeed, we previously found that changes in LAA% and D were greater in patients with, than without a history of exacerbation and demonstrated that assessing the LAA% changes alone cannot determine the coalescence of LAA clusters reflecting parenchymal destruction [17].

We explored which factors would affect changes in D and LAN when LAA% increased in a model that simulated the spatial progression of emphysema in current smokers. In addition to the coalescence of LAA clusters that closely reflects alveolar rupture and leads to a decrease in D [4], [17], we examined whether the decrease in D reflects the spatially heterogeneous disposition of new LAA pixels in the simulation. As shown in Figure 6A, the coalescence of clusters contributed to the decrease in D both in the random and damage-dependent models. Moreover, the damage-dependent model caused a greater decline in D than the random model independently of cluster coalescence.

Consequently, 30% coalescence of clusters was required in the random model to fit the actual reduction in D among current smokers, whereas 15% coalescence was sufficient in the damage-dependent model. We also found that the actual increase in LAN could not be reproduced by the random model at 30% coalescence (Figure 6B). Therefore, the model that leads to spatially homogeneous emphysema progression with intermittent cluster coalescence cannot explain the combination of a decrease in D and an increase in LAN when LAA% increases. This suggests that the progression of emphysema is spatially heterogeneous in current smokers. Although the present model did not directly use mechanics, we speculated that it reflects the possibility that parenchymal destruction can be increased in local regions with severe emphysematous change where mechanical force together with sufficient enzyme activity at the level of septal walls as well as elastin and collagen fibers might be increased and cigarette-smoke induced proteolysis of the extracellular matrix can impair the strength of the alveolar walls [14], [15], [16].

Notably, D was significantly decreased in former smokers during follow-up, while LAA% increased without statistical significance (p = 0.07). This is consistent with the previous finding that emphysema progresses even after smoking cessation [22], [26]. In addition, this is the first longitudinal study to show that D is more sensitive to emphysema progression than LAA%, although a previous cross-sectional CT analysis and network simulation showed that D can be decreased by rupture of the alveolar walls caused by mechanical force even when LAA% does not change; thus D can detect parenchymal destruction more sensitively than LAA% [4].

The variability of lung volumes between baseline and follow-up CT scans could affect lung density measurements [18], [27], [28]. The present study found that the changes in CT-TLV during follow-up were not significant in both current and former smokers, and that the degrees of changes did not significantly differ between the two groups (Table 2 and Figure 4). Furthermore, multivariate regression analysis to adjust for the influence of the change in CT-TLV showed that continuous smoking independently correlated with annual changes in CT parameters of emphysema. This analysis is one of the statistical volume adjustments that can be used to compare CT densitometry among groups of patients, but not to correct individual patient data [17], [18]. Future studies should test whether the present findings can be reproduced by other volume correction methods that have been used to obtain a volume-corrected parameter of emphysema for individual patients [29], [30], [31].

The results of multivariate analysis showed relative contributions of changes in CT-TLV to those in LAA% and LAN, but not to those in D. These are consistent with the notion that lung density parameters such as LAA% can be confounded by changes in lung volume, which might reflect inspiration levels during CT scans and hyperinflation [18], [27], whereas D is probably not affected by changes in lung volume. Figure 4 visually confirms these findings. Our findings strengthen the value of D for detecting progressive emphysema in longitudinal CT studies.

Blood COHb was measured to confirm the validity of self-reporting about smoking cessation. The COHb levels in all former smokers were ≤1.8%, which were far lower than those in current smokers (Table 1). These results were consistent with previous findings [32]. We excluded patients who quit smoking during follow-up because smoking cessation can affect lung density [30]. We believe that this strategy increased the validity of this study.

Although a threshold of −950 HU has been used by others, we used a threshold of −960 HU to calculate LAA% because it has been proven suitable for quantifying emphysema using multi-detector row CT scanners [33], [34].

Some limitations are associated with the present study and one is the small study population. However, we used the same scanner throughout the follow-up period (median 3.57 years) to minimize CT scanner instability. We believe that this overcame the disadvantages of a small sample cohort and generated valid information about emphysema progression.

Secondly, although the aim of the present study was to investigate the effect of continuous smoking on emphysema progression, no current smokers had severe emphysema at the time of the first CT scan (Figure 2). We excluded former smokers with severe emphysema from the analysis to eliminate the possibility that differences in baseline emphysema severity between current and former smokers could be a confounding factor in the comparison of subsequent spatial progression of emphysema between the two groups. Thus, the present results cannot be extended to patients with severe emphysema. Nevertheless, our findings are important because understanding the morphological progression of mild to moderate emphysema induced by cigarette smoke can be helpful, especially for communicating the importance of a smoking cessation strategy during the early stage of the disease.

Thirdly, our simulation was based on the assumption that a framework of lung parenchymal tissue consisting of ND pixels remains fixed when LAA% increases by changing ND pixel into new LAA pixel. This methodology was used in our previous study [17]. In reality, the framework might be altered after lung parenchyma is destroyed. The structure underlying LAA is elastic. Neighboring alveoli might shrink near a larger LAA cluster following an alveolar wall rupture, and then some LAA pixel might change back to ND pixels. This might have also been associated with the finding that D is increased, whereas LAA% remained unchanged in former smokers during follow-up.

However, we assumed that increases in LAA% of up to 3% are so small in the present study that the influence of an altered framework might be negligible. We believe that model simulations using actual CT images in the clinical setting can be applied to many medical issues to deepen understanding of longitudinal morphological changes.

Fourthly, the present model simulation can determine which factors affect changes in D and LAN when LAA% increases. Since LAA% in former smokers did not significantly change during follow-up (Table 2), the simulation cannot be used to interpret changes in former smokers.

In conclusion, our longitudinal CT study revealed that continuous smoking is involved in progressive emphysema assessed as changes in LAA%, D, and LAN. A simulation showed that the increases in LAA% and LAN, and a decrease in D cannot be explained by a model that leads to spatially homogeneous increases in LAAs with the intermittent coalescence of LAA clusters. This suggests that the susceptibility to smoke-induced parenchymal destruction is not uniform over the lung, and thus spatially heterogeneous increases in LAAs occur during progressive emphysema induced by continuous smoking. Our findings support the notion that local regions of relatively advanced emphysematous change are prone to further emphysema progression. Furthermore, the importance of smoking cessation is emphasized and our findings suggest that model simulation using CT images can help to clarify the morphological progression of diseases.
