# Correction: Longitudinal Study of Spatially Heterogeneous Emphysema Progression in Current Smokers with Chronic Obstructive Pulmonary Disease

**DOI:** 10.1371/annotation/793db8a1-2d43-45bd-9ad5-8dac83195b15

**Published:** 2012-11-16

**Authors:** Naoya Tanabe, Shigeo Muro, Susumu Sato, Shiro Tanaka, Tsuyoshi Oguma, Hirofumi Kiyokawa, Tamaki Takahashi, Daisuke Kinose, Yuma Hoshino, Takeshi Kubo, Toyohiro Hirai, Michiaki Mishima

The images for Figures 3, 4, and 5 were incorrectly switched. The image that appears as Figure 3 should be Figure 4, the image that appears as Figure 4 should be Figure 5, and the image that appears as Figure 5 should be Figure 3. The figure legends appear in the correct order. 

